# Can You Hear Me Now? Audio and Visual Interactions That Change App Choices

**DOI:** 10.3389/fpsyg.2020.02227

**Published:** 2020-10-15

**Authors:** Shakthidhar Reddy Gopavaram, Omkar Bhide, L. Jean Camp

**Affiliations:** Luddy School of Informatics, Computing, and Engineering, Indiana University, Bloomington, IN, United States

**Keywords:** usable privacy and security, human factors, visual indicators, audio indicators, audio warnings, android, permissions manifest, resource access warnings

## Abstract

Android and iOS mobile operating systems use permissions to enable phone owners to manage access to their device's resources. Both systems provide resource access dialogues at first use and per-resource controls. Android continues to offer permission manifests in the Android PlayStore for older apps but is transitioning away from this. Neither manifests nor first-use dialogues enable people to easily compare apps based on resource requests, and the corresponding privacy and security risks. Without the ability to compare resource requests when choosing an app, customers cannot select those apps that request fewer resources. Unnecessary and excessive permission requests, overuse of resources, information exfiltration, and risky apps are endemic. To address this issue we built upon past work in warning science and risk communication to design multimedia indicators to communicate the aggregate privacy and security risk associated with an app. Specifically, we provided participants with a privacy rating using the familiar padlock icon and used audio notifications to either warn or reinforce user choices. We empirically tested participants' app decisions with these padlock icons and audio notifications. The results showed that people with both visual cues and audio feedback are more likely to make app choices that are inversely correlated with the resources requested by the app. Those with neither indicators made decisions reflecting only app rating, while decisions made by those with either the audio or the visual indicators are sometimes inversely correlated with resource requests. This illustrates that simple clear communication about apps' aggregate risk, as opposed to atomic resource requests, changes participants' app selections potentially mitigating the state of information overuse and potential abuse. Additionally, neither the visual indicator nor the audio feedback affected the time required for participants to make a decision.

## 1. Introduction

Apps are often over-privileged, asking for more resources and sharing more information than is necessary. For example, Felt et al. analyzed 940 apps and found that one-third of them were over-privileged, meaning that these apps requested permissions for resources that were beyond what was required for the functionality of the app. Apps requested permissions for system calls they could not use and permissions that had been deprecated (Felt et al., 2011). Such over-permissioning can create a risk to both security and privacy. These risks exist even in apps designed for the most vulnerable users, such as those that are designed for children (Reyes et al., 2017).

Users are responsible for managing risks by approving (or disapproving) app permissions requests in both iOS and Android devices. That users are responsible for making these decisions does not mean that they have the ability or incentives to make informed decisions that accurately reflect their preferences. Informed decision-making requires that users understand permissions and their implications. Yet past research has shown that users do not comprehend the permissions much less their implications (Felt et al., 2012; Kelley et al., 2012; Agarwal and Hall, 2013). Additionally, Some risks cannot be determined by resource requests alone; for example, determining which photo app implements editing on the cloud (along with the security of the remote copies) requires focused technical research (Pan et al., 2018). To evaluate apps nontechnical people are relying on peer patterns of use, social feedback, ratings, and Android market reviews. These do not include usable information about over-privileging, use of resources, or corresponding risks.

One approach to mitigate information exfiltration risk is to implement a machine learning model that predicts user preferences and takes appropriate action at runtime (Olejnik et al., 2017). While a machine learning approach can reduce risk by obfuscating or denying access to sensitive resources, it does have some drawbacks. For one, this approach does not address how an app uses the information it collects from the user. For example, once a user provides an app with certain information he/she may not be able to prevent the app from sharing that information with third parties. Additionally, obfuscating techniques may not be effective at protecting user privacy (Shokri et al., 2010), and denying access to certain permissions can render the app unusable. Therefore, a method for communicating risk at the point of sale is still needed to support risk-aware decision-making (Patil et al., 2016). Specifically, it is important to communicate the aggregate privacy risk arising from different sources like permission requests and data usage practices and communicate it to the user at the time of app selection. Such communication of risk at the time of app selection would help participants select privacy-preserving applications while avoiding the above-mentioned issues.

In this paper, we build upon past research in risk communication to design indicators that communicate the aggregate privacy risk to the user at the time of app selection. We provide cognitively simple visual indicators to communicate the aggregate risk associated with an app to address the problem of information asymmetry and user comprehension. We added negative audio feedback to alert users about potentially high risk apps. Similarly, we implemented positive audio feedback for selecting low-risk apps. This audio feedback in combination with visual cues resulted in participants making app choices that are a function of the indicated risk level. We grounded our experiment in previous research on decision-making in psychology as well as in research in warnings and indicators from offline risk communication.

The innovation in this paper is the combination of aural cues and visual icons that prove efficacious in terms of changing decision-making. The goal of this work is to empower users to choose apps based on the implicit risk that is embedded within the app design and resource requests. The underlying assumption is that it is feasible to estimate the risk of an app given the state of art in mobile security and the requirement for apps to explicitly state their resources requests. We provided aural feedback in the form of cheers and jeers in addition to a standard visual icon for security. Not only could participants easily comprehend the positive nature of joyous cheers and the negative implication of angry jeering without any additional cognitive effort, but they are also not interrupted in the app selection task (no additional clicks or screens are needed). Our results showed that participants with both visual and aural cues were more likely to make app choices corresponding to lower risk exposure. The icons, sound files, and JavaScript that implemented the experimental store as well as details on our Institutional Review Board approval are available upon request.

In the immediately following section, we ground our experiment in the existing permissions models, their drawbacks, and the different factors that affect an individual's comprehension of permissions, potential risks, and corresponding decision-making process. Sections 3, 4 give a detailed description of the experiment. Section 5 provides the results and analysis, followed by a discussion of the possible implications of our findings. We close with our conclusion and possible future work with a focus on the interdisciplinary.

## 2. Background

Here we ground our experiment in the user understanding of the permissions models and corresponding potential risks at the time of the work for Android and iOS. We also discuss the implications for the choice of both systems. For both platforms, the two operating systems automatically grant apps permissions to resources that pose very little risk while requiring explicit human interaction to access more sensitive resources. Android has traditionally provided install time permissions manifests. The decision-maker had the option to install the app and grant it all the permissions in the manifest, or they could deny the permissions and not install the app. This is still the case for devices running Android 5.1 or lower. For Android 6.0 and higher, Google is moving toward the more granular run-time iOS model. In the iOS model (and Android versions 6.0 and higher), people are presented with permissions requests during run-time. The first time an app attempts to access a resource (e.g., location), the system generates a resource access warning. These resource access warnings are similar to warning dialogs on other platforms. People also have the option to revoke permissions that were previously granted by navigating to Privacy Settings in iOS or Application Manager in Android. While iOS's model enables setting custom permissions for each app, research has indicated that it fails to provide users the flexibility they desire (Benisch et al., 2011). Prior research has also found that the iOS vetting and run-time warnings were less effective than Android's community ratings and permissions manifest mechanism (Han et al., 2014). A side-by-side comparison of 2,600 apps offered by the same third parties on the two different platforms (e.g., Uber Android vs. Uber iOS) found that the iOS versions consistently access more resources and exfiltrated more data when compared to their Android counterparts (Han et al., 2013). Therefore, expecting the replacement of the Android permissions model with the iOS model to address users' privacy challenges seems unduly optimistic.

### 2.1. Drawbacks of Existing Permissions Models

Neither of the two permissions models has proven to be successful in providing consumers with actionable information for making informed decisions (Agarwal and Hall, 2013). Therefore, both iOS and Android users are largely unaware of the resources accessed by the apps (Mylonas et al., 2013). One of the reasons for this is the users' habituation to ignore the current interactions presented in both Android and iOS permissions models. In the case of textual warnings or permissions manifests used in Android, past research has shown that people usually ignore or pay little attention to them (Felt et al., 2012). More specifically, a series of online surveys and laboratory studies conducted by Felt et al. found that only 17% of the participants paid attention to permissions during app installation (Felt et al., 2012). Consumers are also accustomed to ignoring resource access warnings. Warning dialogs are excessively used in today's computers and mobile devices. This overuse of warning dialogs has desensitized people toward them. Therefore, people view these warning dialogs as interruptions rather than security/privacy alerts and click through them to get on with their current task (Xia and Brustoloni, 2005; Brustoloni and Villamarín-Salomón, 2007; Egelman et al., 2008; Sunshine et al., 2009).

Users' inability to comprehend the permissions presented to them and their implications is another reason why the current permissions models are unsuccessful. Textual warning in permissions manifests, for example, are commonly requested in English with too much technical jargon which effectively assumes that all smartphone users possess an above-average level of basic literacy in addition to computer literacy required to comprehend the permissions information and translate to the risks of agreeing to the requested permissions. However, this is not the case. Not all smartphone users have basic education or computer literacy. As a result, they do not understand the technical jargon used to describe permissions or the implications of providing sensitive permissions to applications (Felt et al., 2012; Kelley et al., 2012). Therefore, even though people value their online privacy (Nissenbaum, 1998), they are unable to make privacy-preserving decisions as the current permissions models fail to provide them with actionable risk information.

In recognition that the previous permissions models were inadequate, there has been a move to automate permissions decisions based on observed user behavior. Models of user preferences may be driven by background observations, possibly augmented by explicit queries about acceptable data use (Olejnik et al., 2017; Wijesekera et al., 2017). Such controls can limit resource use by apps but do not enable apps to compete in the marketplace for risk-averse users. Machine learning mitigates risk, but even those people who value their privacy are unable to make privacy-preserving app selections as there is not adequate decision-making support when needed (Papacharissi and Zizi, 2010). Later automated support to constrain resource use is valuable. Yet, a privacy-seeking user may, for example, accidentally choose a photo or audio app which cannot function without the content being sent to the cloud over a more desirable app unless the information is provided in an easy to comprehend manner at the moment of app selection.

### 2.2. Privacy Indicators

As mentioned above, not everyone has the basic education and computer literacy to understand the information presented in the privacy warning and the risks of giving access to sensitive resources. In such cases, simple privacy indicators that summarize the privacy risks can be beneficial. Locks have been found to have the greatest impact on decision-making in the mobile context (Rajivan and Camp, 2016; Momenzadeh et al., 2020) and communicating security on the web (even when that communication is incorrect; Kelley et al., 2018). Another option for risk indicators, particularly for privacy risk, is the use of eyes as a social cue for information exposure. This has had mixed results. Schlegel et al. (2011) used eyes on the home screen of a smartphone to represent the number of accesses granted to a user's location. The size of the eyes corresponded to the number of times the location was accessed. Liccardi et al. (2014) used eyes to communicate sensitivity score (like our five lock score here) and highlight risky permissions in Android's permissions manifest. Liccardi et al. found that the implicit ranking combined with eyes resulted in significant statistical changes, but he did not compare this with other modes of communication.

Eyes have not consistently proven to be effective or to communicate risk. For example, Benton et al. (2013) compared text with eyes to determine their relative efficacy in communicating aggregate privacy risk to users. Their findings show that eye icons had a stronger statistically significant result when compared both with standard text warnings and brief simplified textual warnings. Yet, using the same eye icons as the previous work, the researchers found that there was no consistent relationship between the impact of the eye icon's effect and the selection of more or less risky apps when roughly accurate ratings were provided using eyes at decision time (Benton et al., 2013).

In a direct comparison between different types of privacy icons in a mobile marketplace, Rajivan et al. studied the effectiveness of three different visual indicators (frowning faces, eye icons, and lock icons), and different framing (positive and negative framing) to evaluate their effect on changes in app selection. The eye icon and face icons were presented with negative framing, as with Liccardi (Liccardi et al., 2014) and Schlegel (Schlegel et al., 2011). The locks were presented as a gain, aka positive framing. The results of the comparison across three icons showed that participants who were presented with positive framing using the padlock made app choices that consistently aligned with increased privacy (Rajivan and Camp, 2016). The impact of the lock icon was significant across all app categories as opposed to the eye icon or the faces. The confidence significantly increased in the presence of priming. Therefore, in our work, we use the lock icons and sought to provide priming with the addition of audio feedback.

### 2.3. Framing of Privacy

Researchers also explored positive and negative framing and how it affected user decisions. Positive framing refers to communicating security as a benefit that is gained rather than security as something that enables loss avoidance. Positive framing is generally supported by work in the psychology of security, although it has been less often applied in the case of mobile marketplaces (Acquisti et al., 2015). West in 2008 identified the underlying human decision-making biases which imply that gain framing would be more effective than loss framing in communicating computing behaviors (West, 2008). Garg expanded on the previous work, focusing on examples comparing loss versus gain framing specifically in computer security (Garg and Camp, 2013). Anderson and Moore (2009) also noted the power of positive framing security information.

In contrast, Choe et al. (2013) initially found limited efficacy for either framing, with little difference between positive and negative framing in an initial study. In a later study, the same authors reified the consensus that the framing of visual cues could affect participants' permissions-based app decisions. That effect was measured by presenting participants with the same app repeatedly and by asking them to make a comparison between two scales (one negative and one positive). The study found that participants made more risk-averse choices with positive framing (Chen et al., 2015).

### 2.4. Timing

Timing also influences user attention to warnings. Balebako et al. investigated the ability of users to recall permissions notices when they were presented under three conditions in the app store: when an app was launched, during app use, and after app use. They used recall as a measure of user attention. Their results showed that people paid more attention to permissions when they were presented during app use (Balebako et al., 2015). Their results also showed that users are unlikely to pay attention to permissions shown in the app store. A difference between that work and ours is that informed decision making, not recall, is the focus of our work.

In contrast, Kelley et al. (2013) found that when permissions were included in the app description page instead of being presented after people chose to install an app, people chose apps that had fewer permissions. In that study, they asked participants to imagine that they were choosing the apps for a friend. We know from risk science that people are more accurate in their risk estimates when making judgments about the acceptability of risk for others. In general, people have been found to be more impartial and risk-averse while recommending a risky situation to others (Helfinstein et al., 2015). Availability, affect, assimilation and representativeness can all result in different estimates for privacy risk for oneself when compared to a friend (Garg and Camp, 2013). Thus, the more risk averse behavior may stem from the experiment design as well as the presentation of permissions. In our study, we used app selections for self, and we minimized the cognitive requirements for our participants by using icons and sound.

### 2.5. Generating Privacy Ratings

Although the generation of accurate Privacy Ratings is not the focus of our research, the possibility of doing so underlies the entire experiment. Therefore, here we provide a shortlist of related work to show that generating such ratings consistently is possible; but not to argue for any of these. Researchers at Carnegie Mellon University have created a website privacygrade.org which gives Android apps a Privacy Grade based on both static code analysis and crowd-sourcing (Lin et al., 2012, 2014). Static code analysis determines what permissions are accessed by an app while the crowd-sourcing aspect determines if the permissions accesses meet user expectations. For example, it is reasonable for Google Hangouts to access a microphone but it would be odd for Angry Birds to do so. It is also possible to rate privacy by analyzing privacy policies. This was demonstrated for websites by Privacy Finder and Privacy Bird (Byers et al., 2004; Cranor et al., 2006; Mcdonald et al., 2009; Tsai et al., 2011). Another promising avenue is the use of natural language processing (NLP) to analyze app description (Pandita et al., 2013). Others have proposed a combination of permission-based risk signals and machine learning techniques to generate a privacy rating (Gates et al., 2014). More thorough evaluations of data flow (e.g., Egele et al., 2011; Pan et al., 2018) and detailed analyses could also be used to develop consistent app ratings (e.g., Beresford et al., 2011; Enck et al., 2011, 2014; Zhou et al., 2011; Arzt et al., 2014).

## 3. Methods and Design

The goal of our work is to see if providing aggregate risk information in form of visual cues (using padlock icons), aural communication, or an integrated warning system containing both would result in users changing their selection of mobile apps. We describe the icons and the sound in detail in this section, grounding them in the previous work from above.

We align our design with the five principles proposed by Rajivan and Camp (2016). Here, we quote directly his conclusions about risk communication. First, “icons should be presented early in the decision-making process while people compare apps to choose and install.” Second, “the scale of privacy communicating icons should be consistent with other indicators.” In this case, the other indicators are rating and download counts. Third, “privacy communicating icons should be in terms of privacy offered by the app/software.” We are evaluating icons for risk, which include privacy and security. Thus we selected a widely used risk communication icon. That we did this is in part based on Rajivan's fourth principle, “icons should align with user mental models of security.” Finally, his fifth recommendation is on requirements for the validity of the underlying rating. This does not apply for this experiment as the risk values are randomly assigned during the experiment to mitigate familiarity issues and more subtle biases from, for example, more attractive app icons.

Much previous work has found that priming for privacy has a significant impact on privacy behaviors, but this priming is not feasible in daily practice (Acquisti et al., 2015). To return to the previous example, Rajivan and Camp (2016) illustrated that the greatest effect in app selection occurred when there was both the lock icon and priming for privacy. Grounded in these findings we used two kinds of interactions: one enables comparisons during app selection and the other functions as a warning or validation before installation. The first is a commonly used visual indicator for security and privacy. It provides a simple and easy way to communicate a summary of risk (e.g., resource requests) across apps in one category. The second, a sound notification as a warning, is also designed to serve to prime users for privacy. Building on the study of hazards and warnings, the icon is intended to provide information processing support while the audio is more aligned with warnings as transmission or alert (Wogalter et al., 2005). The combination of these two messages is designed to create a warning system that addresses both the consumer's right to know (with visual decision support) and the duty to warn (with audio installation warnings) that are at the core of risk communication (Viscusi and Zeckhauser, 1996).

We designed the experiment to measure the effectiveness of the two interactions individually and the combination of them in a warning system. Testing this integrated warning system also requires evaluating each individual component. The control enabled us to compare the discrete components and the entire warning system with previous approaches. In this section, we provide detailed information about the two interactions, the four groups of subjects, and the controlled environment.

### 3.1. Visual Indicator

The goal of the visual indicator is to provide users with easy-to-understand privacy information. A simple icon can ideally inform people with varying levels of literacy. Building upon previous research in this area (discussed in section 2), we employed positive framing using the padlock icon. The design also embeds the standard rubric that when there is a highly variable audience, warnings should be designed for the low-end extreme to include the entire population (Wogalter et al., 2005).

Based on the goal of providing positive framing, more locks imply that an app is associated with lower risk, something that is traditionally indicated through resource requests. In **Figures 2A**, **3A**, we show the lock icon in the context of the *list of apps* page and the *app description* page.

### 3.2. Audio Feedback

The visual icons provide decision support when users are processing information about the apps. The sound provides feedback (a warning or a verification) to the user immediately after selecting an app. The use of sound notifications is both a practical approach to priming and is consistent with the use of tones for creating immediate human responses to potential hazards (Mileti and Sorensen, 1990).

The use of audio in this experiment builds on both warnings research and past human-subjects research in privacy, specifically research involving priming. Users generally make more privacy-preserving decisions when they are primed for privacy, as noted in section 2. However, a common approach to prime for privacy is to use a survey. Questionnaires for app installations in the real world are not workable. Thus we embedded priming in the experiment as an alert consisting of audio snippets of cheers or jeers. The cheers are played when a person selects an app with a high Privacy Rating (privacy-preserving app) and the jeers are played when a person selects an app with low privacy rating (privacy-invasive app). The cheers were intended to encourage people to select more privacy-preserving apps. The jeers, on the other hand, were intended to warn people about privacy-invasive apps.

**Figure 1:**
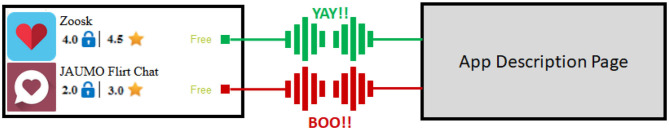
Sounds associated with apps. Choosing an app with a high privacy rating results in cheers and choosing an app with a low privacy rating results in jeers.

We played the audio feedback when a participant selected an app from the *list of apps* page and was transitioning to the *app description* page. An illustration of this is shown in Figure 1. Therefore, these notifications do not create any additional tasks or interrupt the app installation process.

### 3.3. Experimental Groups

To measure how the visual indicators and the audio feedback change users' behavior, we conducted a between-subjects experiment with four experimental groups. There was one control group and three experimental groups: Lock Group, Sound Group, and Warning System Group. The participants in all four groups were presented with a PlayStore simulator which was modeled after Google's PlayStore and simulated the interactions required to install apps on an Android device. However, participants in the experimental groups had additional features available to them. People in the Lock Group were provided with visual indicators for aggregate privacy rating. The participants in the Sound Group heard sound notifications but did not have visual indicators. Finally, the participants in the Warning System Group were provided with visual indicators and were primed for privacy using sound notifications. Table 1 provides the list of features available to each group.

**Table 1:** List of features available in different experimental groups.

**Privacy cues**	**Group 1:** **control**	**Group 2:** **lock**	**Group 3:** **sound**	**Group 4:** **warning system**
Permissions manifest	Yes	Yes	Yes	Yes
Padlock privacy rating	No	Yes	No	Yes
Audio feedback	No	No	Yes	Yes

### 3.4. Experimental Platform

The experimental platform was an interactive PlayStore simulator. Since we are testing aural feedback and decision support to understand the change in behavior caused by the proposed interactions, it is important for us to trigger the decision processes involved in real-world app installations. In To do so, we built an interactive PlayStore simulator modeling Google's PlayStore. The simulator ran on a web browser and provided identical controls and navigation.

**Figure 2:**
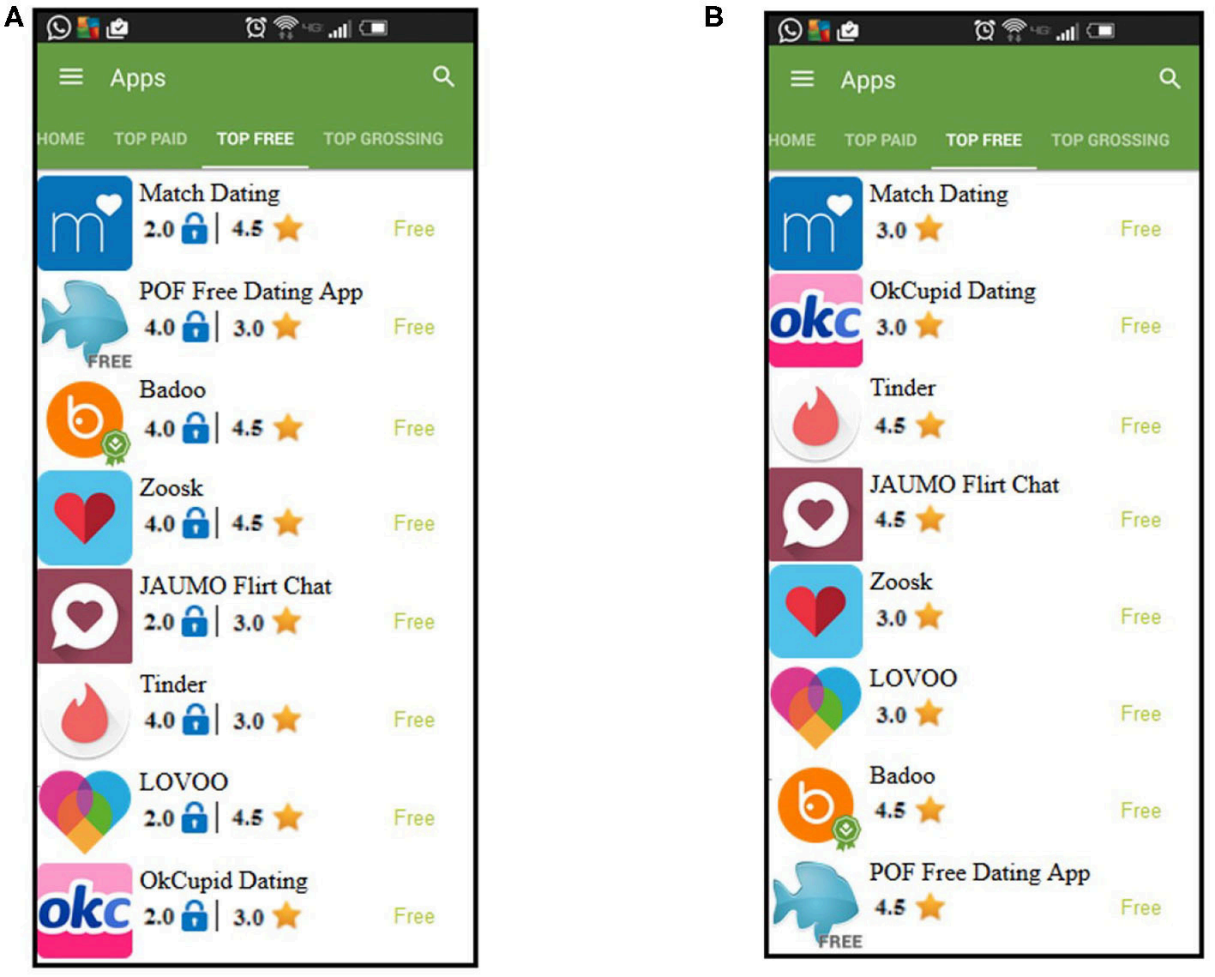
Screenshots of the simulated *list of apps* page. **(A)** For the lock group and warning system group. **(B)** For the control group and sound group.

The simulator consisted of three critical components: the *list of apps* page, the *app description* page, and the flow between them. The *list of apps* page models the interface used by the PlayStore to display apps by category. For this experiment, we produced two versions of the *list of apps* page. One version, shown in Figure 2B, provides users with just the App Rating. This version is used for the Control and Sound groups as participants in these groups are not presented with visual indicators. The alternative version, shown in Figure 2A, augments the *list of apps* page with visual indicators for Privacy Rating in addition to the App Rating. This version is used for experimental groups that provide users with Privacy Rating, i.e., Lock and Warning System groups. In both versions, we only display eight apps per category and when a user selects an app by clicking on it, he/she is redirected to the app description page.

**Figure 3:**
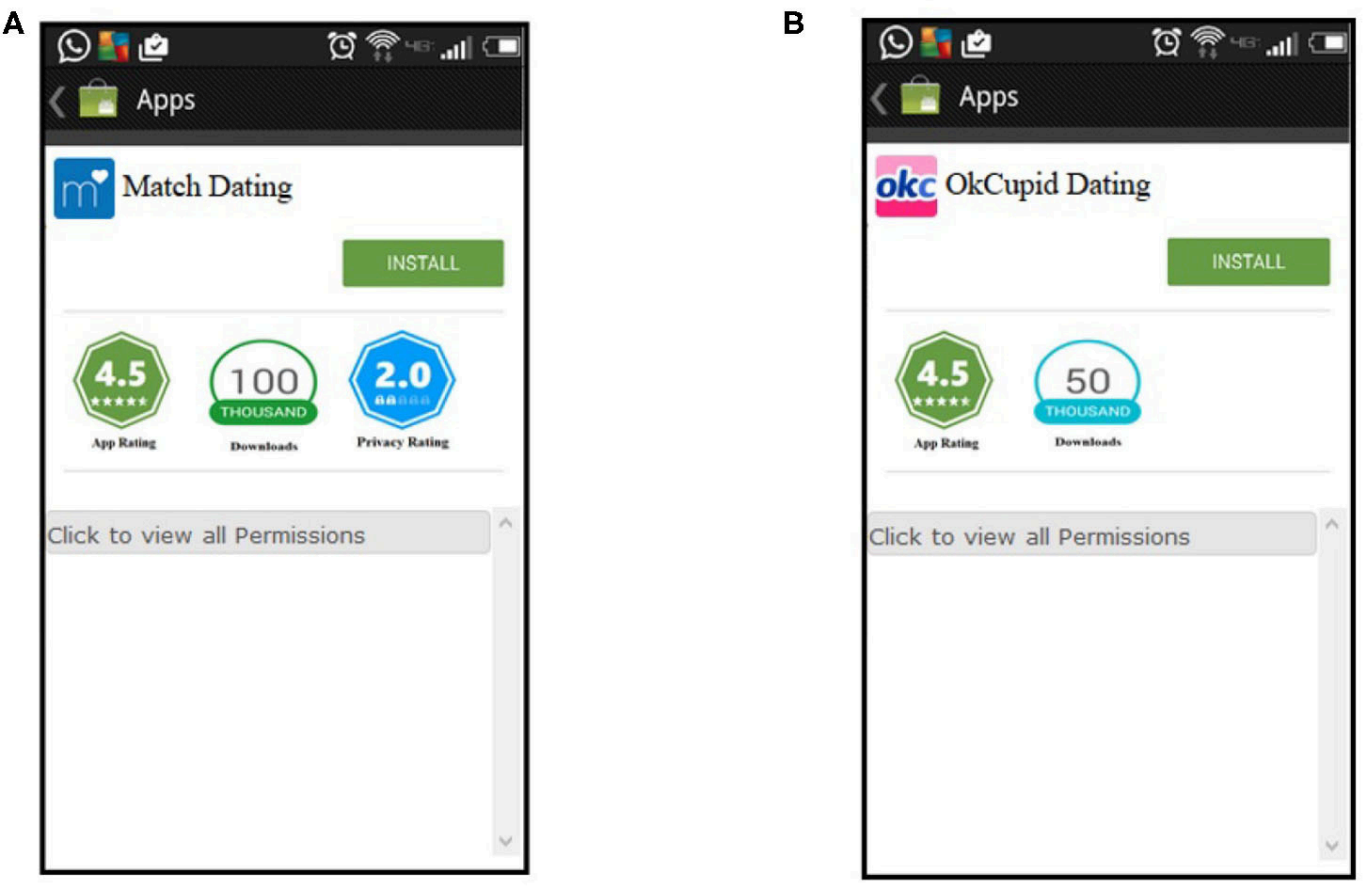
Screenshots of the simulated *app description* page. **(A)** For the lock group and warning system group. **(B)** For the control and sound group.

The app description page on the PlayStore provides users with app rating, download count, a permissions manifest, and an install button. Similar to the *list of apps* page, the *app description* page has two versions: one version with visual indicators for privacy (Figure 3A) and the other without (Figure 3B). The *app description* page without visual indicators for privacy was shown to participants in the Control and Sound groups. The *app description* with privacy visual indicators was shown to participants in the Lock and Warning System groups. For all four experimental groups, clicking on the install button would mimic the installation of the application.

Additionally, for the Sound and Warning System groups, the simulator plays sound notifications after app selection. These sound notifications are played when a user selects an app in the *list of apps* page and is transitioning to the *app description* page. An illustration of this is shown in Figure 1.

All participants were able to navigate the simulator as if in the PlayStore. Specifically, participants were able to move back and forth between the above-mentioned pages using the back arrow, as well as install apps, uninstall apps, and view the permissions manifest by clicking on the *click to view all permissions* dropdown.

### 3.5. Apps

In this experiment, we selected dating and puzzle apps that were popular at the time of the experiment. We derived a total of 16 apps (8 apps per category) from the PlayStore using the top charts filter for each category.

One decision about app selection that varies from previous research is the method of addressing familiarity. Familiarity and reputation are consistently factors in trust decisions in a wide range of online environments (Costante et al., 2015). A series of surveys, interviews, and focus groups illustrated that nontechnical users consistently believe that popularity indicates the acceptability of privacy policies with use by others being an implicit, environmental cue (Morton, 2014). Familiar technologies were found to be perceived as less risky in an investigation of risk perception in mobile and wearable devices (Lee et al., 2015). Specifically, in the case of smartphone applications, past research has shown that users rely on familiarity and majority vote (App Rating) to make app choices (Joeckel et al., 2017). That being the case, it is critical that any interventions introduced to encourage users to make privacy-preserving app choices should be effective in the presence of popular/familiar applications.

**Figure 4:**
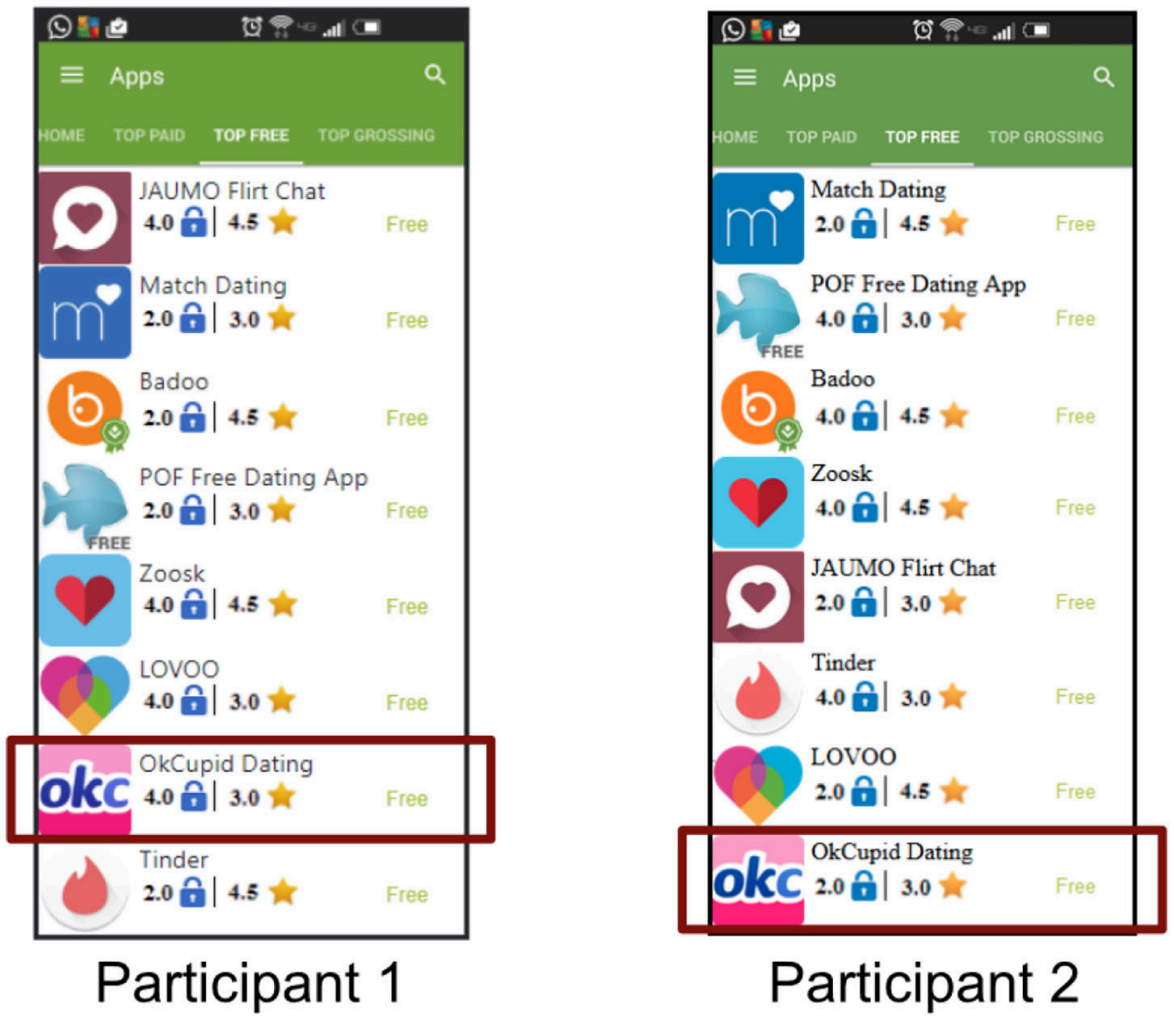
Screenshots of the *list of apps* page for two different participants highlighting randomization of attribute values.

Choosing the inclusion of familiar apps required that the experiment design address the potential bias created by familiarity and reputation. In order to mitigate the biases from familiarity and reputation, we randomized the assignment of values for experimental variables for each and every participant, i.e., the values attributed to the apps will vary from participant to participant. As shown in Figure 4, the Privacy Rating for the OkCupid Dating app is different for participants 1 and 2. Figure 4 shows that seven out of the eight applications have different sets of values for Privacy Rating and App Rating. Therefore, if people keep selecting similar applications because they are familiar with them, then there will not be statistically significant differences between the control group and the experimental groups. We would only find the data to be statistically different if people in the control group make decisions based on different experimental variables when compared to the people in the experimental groups. The difficulty in controlling for familiarity was one reason we choose to recruit a large number of subjects in each category.

We also randomized the order of apps and categories to remove any bias caused by ordering.

### 3.6. Experimental Variables

For each app installed by a participant, we recorded the values for Privacy Rating, App Rating, and Download Count. By recording these values we were able to measure the influence they had on the participants' app choices at the time of app selection. In addition to the three experimental variables, we also compute two other variables PrivacyOverAppRating and PrivacyOverDownloadCount. These two additional experimental variables measure the difference between Privacy Rating and the two remaining variables. In order to compute the values for PrivacyOverDownloadCount, we had to normalize the values for Privacy Rating and Download Count to be on the same scale. So the Download Count values 100 and 50 k would now be 4 and 2, respectively. We then compared the normalized values for Download Count and Privacy Rating against each other. If the Privacy Rating for a selected app was greater than the Download Count then PrivacyOverDownloadCount was assigned to be 1, if Privacy Rating was equal to the Download Count then PrivacyOverDownloadCount was assigned to be 0, and if Privacy Rating was less than the Download Count the PrivacyOverDownloadCount was assigned to be −1. A similar approach was taken to compute the values for PrivacyOverAppRating.

Participants were asked to make 4 app choices per category. This was done to force a situation where it was necessary to make trade-offs between App Rating, Privacy Rating, and Download Count. If asked to make a single choice, participants could optimize across all three variables. By creating multiple choices, we obtain data on decisions where one factor must be chosen over another. In our analysis, we examine the ratio of the three variables to capture the results of these decisions. We choose categories where people tend to make multiple selections, particularly games. People engaged in online dating often also use multiple services (Valkenburg and Peter, 2007).

All three experimental variables were ordinal. For a given app, Privacy Rating(PR) was either 2 or 4, App Rating(AR) took on values 3 or 4.5 and Download Count(DC) was 50,000 or 100,000. We chose to go with higher values for App Rating when compared to the Privacy Rating because extensive past research showed that app ratings dominate choice in the absence of privacy indicators (Kelley et al., 2012; Rajivan and Camp, 2016). Additionally, participants would not want to install an app that is unusable and unwanted, even if it offered the highest privacy. We had adequate variance in app ratings to evaluate this using Generalized Estimating Equations (GEE). Using the values for the three experimental variables, we generated eight combinations of ratings: one app where all the variables had the lowest possible value, one app where all variables had the highest possible value, three apps where only one of the variables had the highest possible value, and three apps where at least two variables had the highest possible value. All eight combinations are listed below.

- Lowest possible values:
  {PR: 2, AR: 3 and DC: 50,000}
- Highest possible values:
  {PR: 4, AR: 4.5 and DC: 100,000}
- One variable with highest possible value:
  {PR: 4, AR: 3 and DC: 50,000}
  {PR: 2, AR: 4.5 and DC: 50,000}
  {PR: 2, AR: 3 and DC: 100,000}
- Two variables with highest possible values:
  {PR: 4, AR: 4.5 and DC: 50,000}
  {PR: 2, AR: 4.5 and DC: 100,000}
  {PR: 4, AR: 3 and DC: 100,000}

As mentioned in section 3.5, these combinations were randomly assigned to eight apps in each category. Requiring users to pick four out of the eight applications means that they cannot optimize all three experimental variables for all four app choices. A participant can at most optimize two variables for two app choices, and for the remaining two choices, he/she can only optimize one experimental variable. This was done to force participants to prioritize one variable over the others.

We also created two example permissions manifests per app category such that one manifest represented over-permissions while the other represented least-permissions. The permission manifest that represented least-permissions was assigned to an app with a high Privacy Rating (4). Similarly, the permissions manifest that represented over-permissions was assigned to an app with a low Privacy Rating (2). This was done to provide internally consistent information. It also enabled privacy-aware participants in the Control Group to distinguish between privacy-persevering and privacy-invasive applications if they viewed the permissions.

In addition to the app choices, we also collected several implicit data measures from the experiment. These were permissions viewed, amount of time spent on choosing apps in each category, and the total time the participants took to complete the experiment.

## 4. Experiment and Participants

The participants for this study were recruited from Amazon's Mechanical Turk (MTurk). Upon agreeing to participate in the study all participants were required to confirm that they owned an Android device. We achieved this by asking participants to visit an URL that provided them with a code only if they visited it using an Android device. Participants were required to have this code to continue with the study. We added this criteria for our study because we wanted to eliminate confounding factors originating from recruiting participants that don't use an Android device. Specifically, past work has shown that people using different platforms have different perceptions about the same app including privacy concerns (Ali et al., 2017; Mcilroy et al., 2017).

Next, all participants were provided with a simple set of instructions on how to use the interactive PlayStore simulator. The instructions were strictly mechanical, explaining that the participants had to select apps. After reading the instructions, the participants were allowed to progress to the simulated environment and make app choices. They were presented with two sets of app categories with eight apps in each category. After selecting the applications, participants answered demographic questions and questions for consistency checks. The order of categories, the order of apps under each category, and the ratings (Privacy Rating, App Rating, and Download Count) assigned to the apps were randomized for all participants. The categories were dating apps and puzzle apps.

Participants were asked to make four app choices in the order of their preference for each category, with the first choice being the most preferred and the fourth choice being the least preferred. Once the participants made all the necessary app choices, they were presented with queries about their app installation behavior, their computer literacy, and their demographics.

Reproductions of classic experiments have shown that the response of MTurk participants to priming and framing is consistent with participants in laboratory and field experiments (Horton et al., 2011). The use of MTurk is appropriate for this controlled study based on previous research and accepted practice (Horton et al., 2011; Casler et al., 2013; Chong et al., 2017). In methodologically validating related work conducted by Casler et al., participants were presented with four pairs of tools and they had to pick one tool from each pair to perform a task (Casler et al., 2013). While the in-lab participants were allowed to physically hold the tool, the Mturk participants only saw demonstrations of the tool being used. The researchers compared results from the laboratory study to that of the online simulation conducted on Mturk and found that the results were indistinguishable. In our work, participants perform the same actions to install or uninstall an application (the simulator replicated the interactions that users performed on the PlayStore) with a different mode of interaction (mouse vs. touch).

## 5. Results

In the following, we begin with a rough summary and visualizations of the results. Then we provide a detailed statistical analysis.

### 5.1. Demographics

The study features four groups of subjects with three variables in each. Eighty participants were recruited for each experimental condition. In total, we enrolled 320 participants for our study. This was larger than the number required by power analysis by more than a factor two.

Out of the 320 participants, 17 participants were disqualified for providing contradicting answers to questions in the questionnaires. For example, the question “Do you review/read the permissions presented to you before you install an app from the Google PlayStore?” was asked twice. Participants that gave two different answers were disqualified. We also excluded all the results from the participants who took <3 min to complete the study. After applying the above mentioned exclusion criteria, we ended up with a total of 235 participants. These exclusion criteria were used to identify participants who only put minimal effort toward making app choices. We then repeated the analysis without excluding those who took <3 min; the results were stronger in that there were smaller *p*-values. However, here we include the analysis for the smaller sample as our initial study design included the 3-min-limit.

We applied a location qualification in MTurk to require all participants to be within the United States. Out of the 235 participants, 60.85% were male and 39.15% were female. The majority of the participants were 25–35 years old (50.21%). 23.4% of the participants were between 35 and 45 years old, 14.8% were 18–25 years old, and 11.4% were older than 45. We cannot argue that the sample was representative of the U.S. population as a whole. Other scholars have noted that MTurk use limits representativeness and participation (Stritch et al., 2016). Conversely, MTurk is widely used and thus these results can be compared to similar related work, with multiple studies indicating that MTurk is a reliable resource for high-quality data (Buhrmester et al., 2016).

### 5.2. Basic Means Comparison

**Figure 5:**
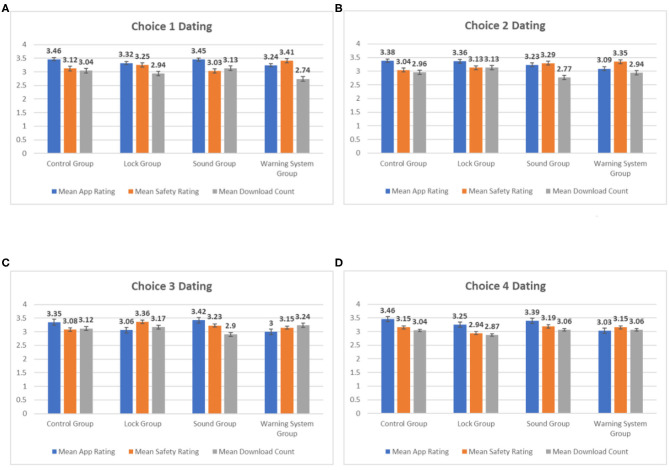
Mean values for dating apps. **(A)** Means for app choice 1, **(B)** means for app choice 2, **(C)** means for app choice 3, and **(D)** means for app choice 4.

Figure 5 shows the histograms of mean App Rating, Privacy Rating, and Download Count for the four app choices in the dating category. As you can see in Figure 5 the mean App Rating for all four choices in the control group is higher than the mean Privacy Rating and the mean Download Count. This indicates that participants in the Control Group were seeking a higher App Rating rather than maximizing Privacy Rating or Download Count. In contrast, the mean Privacy Rating is consistently higher than the mean App Rating and Download Count in the Warning System Group. Choice 3 is the only exception [Mean Download Count (3.24) is greater than Mean Privacy Rating (3.15)]. The mean Privacy Rating of the Warning System Group is higher than the mean Privacy Rating of the Control Group for the first three app choices. The mean Privacy Ratings for the fourth app choice are the same for both groups, but it is roughly equal to the App Rating. The Lock Group and the Sound Group also consistently showed a higher mean Privacy Rating when compared to the Control Group. Choice 1 is an exception for the Sound Group [Control Group (3.12) > Sound Group (3.03)] and choice 4 is an exception for the Lock Group [Control Group (3.15) > Sound Group (2.94)]. This shows that the Privacy Rating of the apps was higher when the participants were provided with the privacy cues. The trends are particularly clear in the Warning System Group.

**Figure 6:**
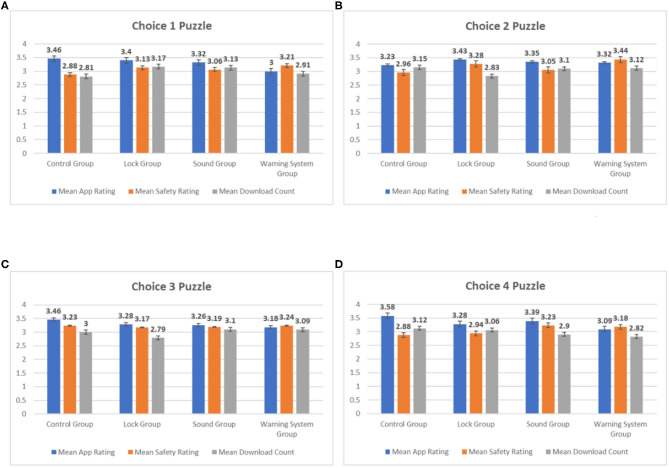
Mean values for puzzle apps. **(A)** Means for app choice 1, **(B)** means for app choice 2, **(C)** means for app choice 3, and **(D)** means for app choice 4.

Figure 6 shows the histograms of mean App Rating, Privacy Rating, and Download Count for the four app choices in the puzzles category. Similar to the dating apps, the mean App Rating for all four app choices in the Control Group is higher than the mean Privacy Rating and Download Count, indicating that participants' in the Control Group made their app choices that optimized App Rating. One other similarity is that the mean Privacy Rating for all four choices in the Warning System Group is higher than the mean App Rating and Download Count. This indicates that Privacy Rating had more influence on the app choices made by the participants in the Warning System Group when compared to the Control Group. This implication is strengthened by the fact that the mean Privacy Rating for the Warning System Group is higher than that of the Control Group for all four app choices. Also similar to the dating apps, the mean Privacy Ratings for the Lock Group and the Sound Group are higher than that of the Control Group for three out of four app choices [mean Privacy Rating Control Group (3.23) > mean Privacy Rating Sound Group (3.19) > mean Privacy Rating Lock Group (3.17) for Choice 3]. Privacy Rating appears to have had more influence on app choices made by participants in groups with privacy cues when compared to the Control Group. Once again, this trend is most prominent in the Warning System Group.

### 5.3. Analysis

Typically, to determine if the difference between groups is statistically significant, a researcher would perform one-way Kruskal–Wallis and pairwise Mann–Whitney (pairwise comparison) tests for non-parametric data. These are commonly used to determine statistical differences between groups and are often requested by reviewers. However, in order for these tests to generate accurate results, the data must conform to certain assumptions. These assumptions are as follows:

- The dependent variable must be measured on an ordinal or continuous scale.
- The independent variable (in our case Groups) should have two or more categories.
- The observations must be independent (i.e., there should be no relationship between observations in each Group or between Groups).

Our study data violates one of the three assumptions. The recorded observations are not independent i.e., each participant makes four app installation choices which results in a dataset where the dependent variables (App Rating, Privacy Rating, PrivacyOverAppRating, PrivacyRatingOverDownloadCount) are correlated. If these correlations are not taken into account the results from the statistical analysis will not be valid and the results will be non-replicable. Therefore, to accurately determine the statistical differences between the control group and the experimental groups, we used Generalized Estimation Equations which requires no such assumptions.

#### 5.3.1. Generalized Estimation Equations

Generalized Estimating Equations (GEE) are an extension of Generalized Linear Models and are commonly used to analyze correlated data that arises from repeated measurements (Hardin, 2005; Seago et al., 2006; Lee et al., 2007; Muth et al., 2016). In our case, the repeated measurements stem from each participant making four app installations in each category. A GEE analysis can evaluate the aggregate decisions to see if users in different groups behaved differently. GEE does not restrict the dependent variables to be continuous or require normal distribution. GEE aligns with our experimental goals and the resulting data.

When reporting the results from our analysis we provide both the p-value and the odds ratio. The p-value indicates the strength of the evidence against the null hypothesis and the odds ratio provides an effect size. The odds ratio represents the odds that an outcome will occur given a particular exposure compared to the odds of the outcome occurring in the absence of the exposure. In our case, the odds ratio is interpreted as follows. When the *Odds ratio* = *1* this implies that the cues in the experimental group do not affect the outcome. When the *Odds ratio* > *1* this indicates that participants in the experimental group are likely to have a higher value for the given dependent variable. When the *Odds ratio* < *1* this indicates that the participants in the experimental group are likely to have a lower value for the given dependent variable.

#### 5.3.2. Privacy Rating, App Rating, and Download Count

We performed GEE analysis on the collected data to see if Privacy Rating, App Rating, and Download Count were significantly different between the control group and the experimental groups. In this section, we report the results of our analysis.

**Figure 7:**
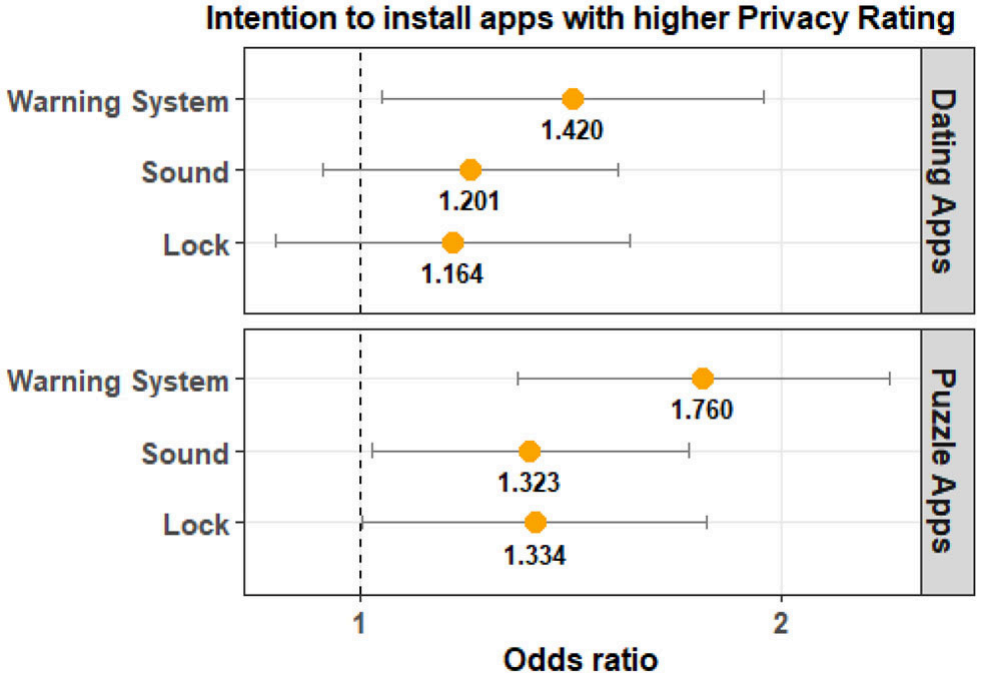
The odds ratio (95% confidence interval) indicates that participants in the Warning System Group are more likely to select apps with a higher Privacy Rating compared to the Control Group.

The results for Privacy Rating are shown in Table 2. These results indicate that Privacy Rating is not significantly different from that of the Control Group for both Lock and Sound groups across the two app categories. For the Warning System Group, Privacy Rating is statistically significant for puzzle apps and marginally significant for dating apps. The odds ratio indicates that participants in the experimental groups are more likely to choose an app with a higher Privacy Rating when compared to that of the Control Group. Participants in the Warning System Group are 1.42 times more likely to select a dating app with a higher privacy rating and 1.76 times more likely to select a puzzle app with a higher privacy rating when compared to the Control group. The magnitude of the effect is clearly higher for puzzle apps when compared to dating applications. A visualization of this is provided in Figure 7.

**Table 2:** For the Warning System Group, the results are significant for puzzle apps and marginally significant for dating apps.

		***p*-values**	**Cohen's d**
Warning system group	Dating apps	0.059	0.193
	Puzzle apps	0.001	0.312
Lock group	Dating apps	0.264	0.084
	Puzzle apps	0.063	0.159
Sound group	Dating apps	0.146	0.101
	Puzzle apps	0.063	0.154

*For the remaining two experimental groups the results are not significant for both app categories*.

**Figure 8:**
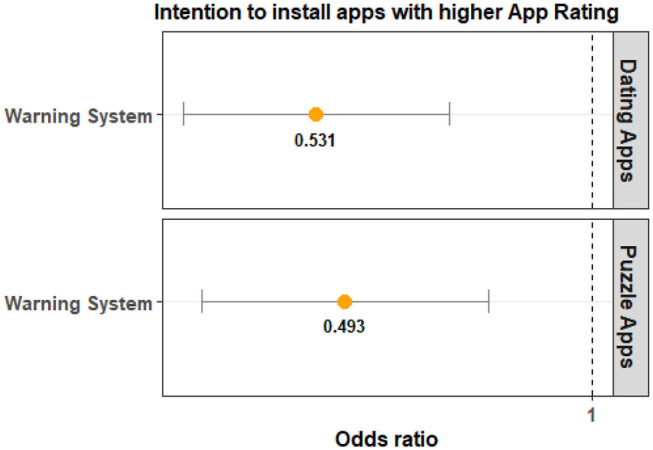
The odds ratio (95% confidence interval) indicates that participants in the Warning System Group are more likely to select apps with a lower App Rating compared to the Control Group. Warning System is also consistently significant for both app categories.

The results from the analysis on App Rating can be found in Table 3. The results show that App Rating is statistically significant for the Warning System Groups across both app categories. For the Lock and Sound groups, the results are not statistically significant. The visualization for the odds ratio is shown in Figure 8 shows that participants in the Control Group are more likely to select an app with a higher App Rating when compared to the Warning System Group. This effect was larger for dating apps when compared to puzzle apps.

**Table 3:** The *p*-values show that App Rating is significantly different for the Warning System Group for both app categories.

		***p*-values**	**Cohen's d**
Warning system group	Dating apps	*p* < 0.001	−0.349
	Puzzle apps	0.002	−0.390
Lock group	Dating apps	0.074	−0.210
	Puzzle apps	0.285	−0.109
Sound group	Dating apps	0.465	−0.056
	Puzzle apps	0.179	−0.122

*For the Lock Group and the Sound Group the results are not significant*.

Download Count was not found to be significant for all three experimental groups.

#### 5.3.3. PrivacyOverAppRating and PrivacyOverDownloadCount

To understand the impact Privacy Rating had on the participants' app choices in comparison to App Rating and Download Count, we examined the ratio of Privacy Rating to App Rating as well as Privacy Rating to Download Count. To be more descriptive, we performed GEE analysis on the dependent variables PrivacyOverAppRating and PrivacyOverDownloadCount. As discussed in section 3.6, PrivacyOverAppRating tells us if the Privacy Rating for an installed app is greater than (1), equal to (0), or less than (−1) its App Rating. Similarly, PrivacyOverDownloadCount tells us if Privacy Rating for an installed app is greater than (1), equal to (0), or less than (−1) its Download count. A higher value for PrivacyOverAppRating or PrivacyOverDownloadCount indicates that participants attributed more weight to Privacy Rating at the time of app selection relative to App Rating and Download Count.

**Figure 9:**
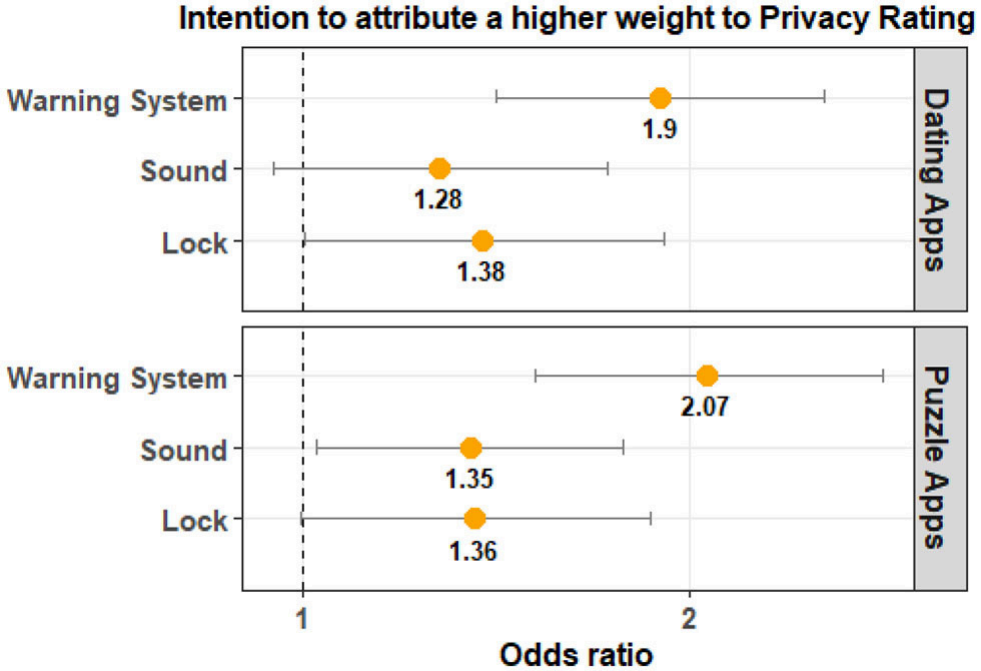
The odds ratio (95% confidence interval) illustrates that participants in the Warning System Group are more likely to choose an app with a higher value for Privacy Rating relative to App Rating. Warning System is also consistently significant for both app categories and has a higher odds ratio compared to the Lock and Sound groups.

Table 4 shows that the PrivacyOverAppRating is significantly different between the Control Group and the Warning System Group for both app categories. The odds ratio tells us that participants in the Warning System Group are more likely to have a higher PrivacyOverAppRating value when compared to the Control Group. This implies that Privacy Rating had a larger impact on the users' app choice when compared to App Rating. Once again the magnitude of the observed effect was larger for puzzle apps when compared to dating apps. The odds ratio and the visualization of the comparison can be seen in Figure 9.

**Table 4:** The *p*-values indicate that PrivacyOverAppRating is significantly different for the Warning System Group for both app categories.

		***p*-values**	**Cohen's d**
Warning system group	Dating apps	*p* < 0.001	0.354
	Puzzle apps	*p* < 0.001	0.401
Lock group	Dating apps	0.063	0.178
	Puzzle apps	0.072	0.170
Sound group	Dating apps	0.150	0.136
	Puzzle apps	0.059	0.166

*For the Sound Group, the results are marginally significant for puzzle apps*.

For the Lock Group, the results were not statistically significant for both app categories. For the Sound Group, the results were not significant for dating apps and were marginally significant for puzzle apps. The odds ratio indicates that PrivacyOverAppRating is likely to be higher for the Lock Group and Sound Group. As shown in Figure 9, the magnitude of the effect is larger for the Warning System group.

**Figure 10:**
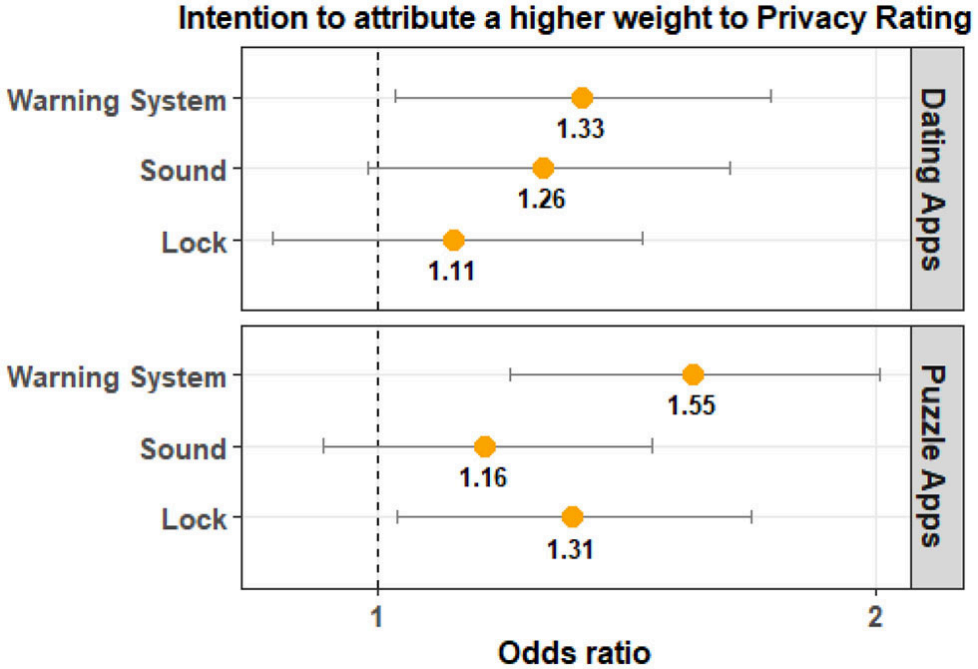
The odds ratio (95% confidence interval) illustrates that participants in the Warning System Group are more likely to choose an app with a higher value for Privacy Rating relative to Download Count.

The results for PrivacyOverDownloadCount are shown in Table 5. For the Warning System Group, the PrivacyOverDownloadCount is statistically significant for puzzle apps and marginally significant for dating apps. The results are not significant for the remaining two experimental groups. The odds ratio shows that the value of PrivacyOverDownloadCount is likely to be higher for the Warning System group. Similar to other instances, the magnitude of the effect is larger for the puzzle apps when compared to dating apps (see Figure 10).

**Table 5:** For the Warning System Group, the results are significant for puzzle apps and marginally significant for dating apps.

		***p*-values**	**Cohen's d**
Warning system group	Dating apps	0.059	0.157
	Puzzle apps	0.002	0.242
Lock group	Dating apps	0.329	0.058
	Puzzle apps	0.063	0.149
Sound group	Dating apps	0.074	0.127
	Puzzle apps	0.146	0.082

*For the Lock and Sound groups the results are not significant*.

To summarize, the Warning System Group is significantly more likely to have a higher value for both PrivacyOverAppRating and PrivacyOverDownloadCount than the Control Group. From this analysis, we can argue that participants with both visual and aural cues are more likely to make decisions reflecting a relatively higher attention to Privacy Rating.

#### 5.3.4. App Installation Frequency

The efficacy of aural feedback may be a function of its novelty. Audio feedback in this work was implemented both as a form of priming, and for the negative sounds, as a warning. Excessive use of visual dialogs has desensitized people's awareness of security warnings on the web (Anderson et al., 2016; Vance et al., 2017). At the end of the survey, we asked participants how often they installed apps from Google's PlayStore. No one reported that they never installed apps from the app store. Respectively 15, 32, and 40% reported installing apps every other month, monthly, or weekly. A median user would see the warnings more often than once a month, and less often than once a week. The remaining participants reported that they installed apps on average every other day (9%), daily (2%), or more than once a day (also 2%). On average users would interact with the warnings every twenty-three days assuming thirty-day months. Habituation cannot be dismissed as a threat for all users, especially the 13% that would see the warning every other day or more. However, since 87% of the participants reported that they installed apps from the PlayStore *once a week* or *less often than once a week*, this indicates that for a large population habituation may be less of a concern. By definition, warning on first use only applies when a new app has been installed and is first run, app installation is an activity that does occur at roughly the same frequency as the first run or somewhat more often. Also note that, unlike warning dialogs, the specific audio feedback is unique and is not used by other computing devices. It is worth considering that our feedback does not interrupt the task flow. There is no dialog to close in this interaction, so this makes the communication potentially more acceptable it may also be easier to ignore over time.

#### 5.3.5. Time to Decision

To determine if the addition of sound to the interaction was overwhelming, we compared the *time to decision* by participants in each condition. To further measure if the decision-making was burdensome, we conducted one-way ANOVA to test the differences of mean decision times between experimental groups. The differences in the means were not significant (*p*-value = 0.269). The mean times were 1.729, 1796, 1.760, and 1.859 for Control, Lock, Sound, and Warning System groups respectively. Previous work which compared different Internet panels for quality of data indicated that time to complete a survey was correlated with quality of data, and thus the decision to curtail participants by time to completion (Smith et al., 2016).

## 6. Discussion

As mentioned in section 5.3, the results show that people provided with both visual indicators and aural feedback are more likely to select apps with a higher Privacy Rating. This finding aligns with studies of warning systems offline, where information processing support impinges decision-making, and aural feedback is the most effective mode of communication at the time of exposure to a potential hazard.

In our study, we utilized attention check questions and time taken to install apps to identify and filter out participants who responded in an inattentive fashion. While attention check questions are known to be effective at identifying inattentive responses, response times were found to be unreliable for identifying inattentive responses (Downs et al., 2010; Gadiraju et al., 2015). The ineffectiveness of completion time as a filter could be due to the noise added by variability in computer load time, mouse maneuvering, and differences in cognitive processing time (Downs et al., 2010). Additionally, past research has shown that participants gaming the system use different strategies and take varying amounts of time (Gadiraju et al., 2015).

The decision to reconsider time as a variable was also influenced by the effect of attitudes on decision-making time (Fazio et al., 1989). Those familiar with the apps may have lower decision latency.

So it is not possible to separate the inattentive participants using completion time. As app installation time as a filter was a part of our initial study design, we reported results for participants who passed both attention checks. Since response time is now not considered a reliable method to filter out inattentive participants, here we report a subset of the results for all participants that passed the attention check questions without filtering out participants for app installation time. The complete results can be found in the Appendix.

**Figure 11:**
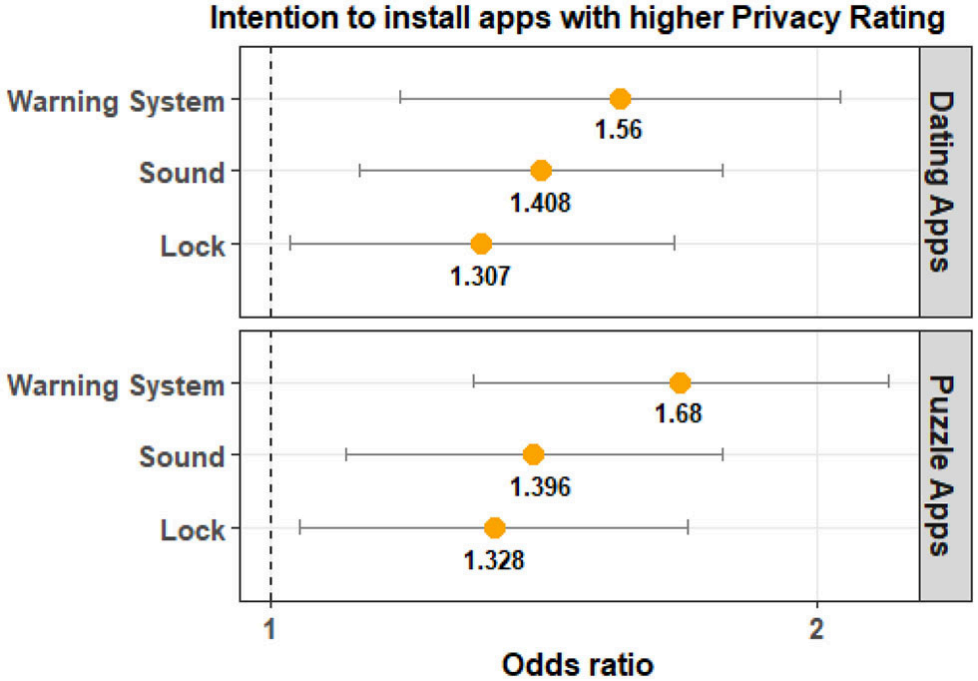
The odds ratio (95% confidence interval) indicates that participants in all three experimental groups are more likely to select apps with a higher Privacy Rating. The effect size is larger for participants in the Warning System Group.

Table 6 shows the results from the statistical analysis of data without the time filter for Privacy Rating. These results have been adjusted for multiple testing. The results show that Privacy Rating was significantly different from the Control Group for all experimental groups. The odds ratio indicates that participants in all three experimental groups are more likely to select apps with a higher Privacy Rating. The effect size is larger for participants in the Warning System Group. This is illustrated in Figure 11.

**Table 6:** GEE results for Privacy Rating for data without the time filter with adjustments for multiple tests.

		***p*-values**	**Cohen's d**
Warning system group	Dating apps	0.001	0.245
	Puzzle apps	*p* < 0.001	0.286
Lock group	Dating apps	0.010	0.148
	Puzzle apps	0.009	0.156
Sound group	Dating apps	0.002	0.189
	Puzzle apps	0.003	0.184

*These results show that participants in all experimental groups made app choices that are significantly different from that of the control group*.

The differences in results when decision time is not a filter indicate the potential for more research on how attention, decision time, and even distraction affect the efficacy of cues and warnings. These results show a clear significance for the Warning System across both categories. Sound is strongly significant for dating and puzzles; while Locks are similarly significant for both.

Under the most stringent analysis participants who were presented with only visual indicators or only audio feedback were not statistically different from the Control Group. This indicates that when people are presented with only visual indicators or audio feedback for privacy, they may not consistently make app choices that are privacy-preserving. This explain the inconsistent findings about privacy cues in previous work. This finding argues for more nuanced investigations on nudging privacy decision-making.

When the Privacy Rating was provided alongside the App Rating using only icons or only sound, we can not be entirely confident that participants' decisions were affected by the Privacy Rating. Without the audio feedback priming or warning participants to consider the Privacy Rating, they were less likely to pay attention to the visual cue. Conversely, when participants are provided with audio feedback but no visual indicator for Privacy Rating, then they may not be able to understand the implications of the audio feedback. As this is the first study on audio feedback in mobile resource warnings, more studies are needed to evaluate the efficacy of different sounds, or similar sounds with a different tone, pitch, and volume.

One possible reason for the disparity between the app choices for dating and puzzle apps could be that participants were more willing to share sensitive information with dating apps when compared to puzzle apps. It is clear why a dating app would require access to sensitive resources. For example, it is easy to understand that a dating app requires access to users' location to find people around them. But the same cannot be said about puzzle apps or game apps in general. For example, in a study conducted by Shklovski et al. participants felt deceived and expressed concerns when they learned about data collected by the Fruit Ninja app (Shklovski et al., 2014). In Lin et al. (2012), crowd-sourcing found that the acceptability of the same permissions varied across different apps.

Finally, regardless of cues, download count information was not significant in the app decision making process. Part of the reason could be that the download count values used for the experiment were not sufficiently different to influence app choices. Another reason could be that findings which indicate that download count dominates decision processes may have been observing a hidden variable (for example, the order of presentation or familiarity). We included the results for download counts in our paper because the lack of impact of download counts on participants' app choices is a significant finding even if it is only for relatively a smaller difference in download count. More research is needed to understand if larger variances in download count affect participants' app choices.

Our results indicate that participants who engaged with a multimedia warning system were more likely to make privacy-preserving app choices than those provided only with audio feedback or visual indicators. Consistent user awareness of privacy risks could have a significant cumulative effect on the entire mobile ecosystem. Given that one person's privacy choices impinge on the privacy of that person's contacts and potentially even those who share local area networks or physical location, a small but consistent improvement in mobile resource use by apps could have significant effects.

One further area of investigation is the relationship between fear and aural warnings. If the warnings create a fear response, this would be correlated with an increase in security behaviors (Johnston and Warkentin, 2010). In this case, the aural warning would have increased perceptions of privacy as a threat and decisions would be impinged by perceptions of self-efficacy and the efficacy of the response. Extensions of warnings research that includes protection-motivation theory and how behavior is impinged by fear could contribute to a more nuanced understanding of app selection behaviors (Herath and Rao, 2009).

Did these function as warnings to which users would become habituated or did they provide decision support that would remain valuable? Since past research has shown that people are less likely to become habituated to polymorphic warnings (Anderson et al., 2016; Vance et al., 2017) an evaluation of polymorphic aural warnings would be worthwhile. *In-situ* experiments that measure user behavior in the complex real world, without the focus here on isolating experimental variables in our controlled study, would be ideal. There is also a need for deep qualitative investigations of the privacy perspectives of end-users. Both *in-situ* evaluations and qualitative investigations should include participants with varying levels of privacy preferences and technical expertise.

## 7. Conclusion

Our experiment tested the efficacy of a visual cue, audio feedback, and a combination of these. We grounded this in usable security and were informed by heuristics from warning science. We provided padlocks as a visual privacy cue in the presence of a realistic distribution of apps both with and without audio feedback. We considered other options (such as haptic interactions and additional visual framing) for priming users for privacy. We chose audio feedback because haptic interactions are not clearly good or bad, and additional visual framing could be confounding or interrupt the task. Audio warnings also have been found to be effective in creating immediate awareness of physical hazards, and some effect was also seen here.

The results from our experiment showed that when participants were presented with both visual (positively framed padlocks) and aural indicators (cheers and jeers), they made app choices that included consideration of privacy ratings; i.e., individuals chose apps with higher privacy ratings over apps with higher app ratings. This was a significant change in behavior when compared to the Control Group, where participants made app decisions primarily based on app ratings. Reflecting on the body of previous research, those participants who saw only icons did not consistently make decisions that were correlated with higher app ratings. Hence, the inclusion of aggregate ratings and multimedia priming offers promise for supporting more informed decision making in online app stores. An added benefit of the approach we present here is that it could create competition or incentives to develop apps that are more conservative in terms of permission use. Currently, many apps are over-privileged perhaps in part because there is little to no marketplace benefit to minimizing permissions requests.

One of the limitations of our study is that we don't compare paid apps against free apps. However, we note that past work examined free Android apps and their paid counterparts, and showed that there is no evidence to support that the premium versions of the same app offered more privacy when compared to their counterparts (Han et al., 2019). Additionally, the current payment structures are based on monetization strategies, maintenance costs, and features not privacy (Ali et al., 2017).

These are promising results, yet additional research is indicated before the model of audio feedback and visual cues are accepted as ground truth. One area of future research is how to distinguish between two apps that have two different but close privacy ratings, for example between 2 and 2.3. This would suggest the use of a continuous sound variable, ranging from intensely negative to strongly positive. Such future work could be informed by a participatory design approach, as this offers promise in evaluating how different audio indicators may convey privacy information. This method may be particularly useful for the identification of continuous instead of discrete sound options. While this research was focused on detecting effects among the participants from the MTurk population, it is worth noting that screen readers do not consistently read nor report security indicators. Thus another avenue of future work would include the visually impaired.

Longitudinal investigations could determine if these effects are a result of a lack of familiarity or improved decision support.

## Data Availability Statement

Subject only to approval by the Institutional Review Board for data anonymization, the datasets generated for this study are available on request to the corresponding author.

## Ethics Statement

The studies involving human participants were reviewed and approved by Indiana University IRB. The patients/participants provided their informed consent to participate in this study.

## Author Contributions

SG: worked on building the play store simulator (experimental environment), contributed to study design, conducted the Mturk study, and performed data analysis. LC: principle investigator, and contributed to study design. OB: worked on building the play store simulator (experimental environment), and contributed to study design. All authors contributed to the article and approved the submitted version.

## Conflict of Interest

The authors declare that the research was conducted in the absence of any commercial or financial relationships that could be construed as a potential conflict of interest.
