# Supplementary material for: Can You Hear Me Now? Audio and Visual Interactions That Change App Choices
Source: Front Psychol. 2020 Oct 15;11:2227. doi: 10.3389/fpsyg.2020.02227 (PMC7593676; doi:10.3389/fpsyg.2020.02227)
Supplement: Supplementary file 1 [file Data_Sheet_1.pdf]

# *Supplementary Material*

## 1 Instructions

**Device Verification**: Please visit the following URL (url presented here) on your Android phone and enter the verification code in the text box below.

If you do not have access to an Android phone or your browser does not support javascript. Please release this task to allow other people to participate and to avoid a rejection.

**Instructions (Please read carefully before proceeding. Read more than once if needed)**

Next you will be shown a simulation (imitation) of the PlayStore such as this (Figure S1).

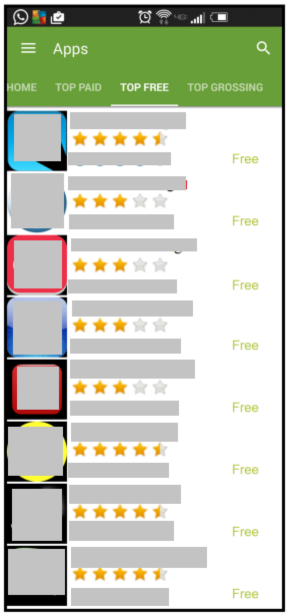


**Figure S 1.** List of apps figure shown in instructions

As you can see, the above screen presents a list of applications belonging to an application category (Ex category: Games)

This is similar to the interface/screen that you would see when you visit the Android PlayStore to install applications for your phone.

To select an application, you have to click on the image of the application.

Here you will be asked to review the applications presented and choose at least 4 applications that you would like to install.

You will install the application in the same way as you would on your android mobile phone. You can also uninstall an application that you have installed.

Make 4 application choices in the order of your preference such that the first choice is the most preferred and the last choice is the least preferred.

There will be an indicator, similar to the ones shown below, at the top of the page. It will help you keep track of the number of applications you have installed.

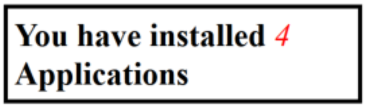


**Figure S 2.** Indicator for participants to track number of installed apps

After you make 4 application choices for a category, you will be shown a “Continue to next App set” button (shown below). Clicking on this button will take you to the next application category

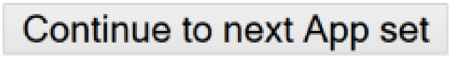


**Figure S 3.** Continue button

You will be presented with 2 categories of applications: Puzzle apps and Dating apps. Remember you cannot go back and change the choices you made once you proceed from an application category to the next.

Reading these instructions more than once could help you avoid any confusion. Please try to enjoy the task.

That’s it! Sit back, relax and simply install applications on the PlayStore simulator.

# 2 Questionnaires

## 2.1 Expertise

1) Have you ever (select all that apply)
   a) Designed a website
   b) Registered a domain name
   c) Used SSH
   d) Configured a firewall
   e) Created a database
   f) Installed a computer program
   g) Written a computer program
   h) None of the above

2) Do you have a degree in an IT-related field (e.g., information technology, computer science, electrical engineering, etc.)?
   a) Yes

b) No

3) Have you ever taken or taught a course on computer security?
   a) Yes
   b) No

4) Is computer security one of your primary job responsibilities?
   a) Yes
   b) No

5) Have you ever attended a computer security conference in the past year?
   a) Yes
   b) No

6) Please indicate which of the following indicators you use to decide if it is safe to enter your username and password on a particular website.
   a) https
   b) website certificate
   c) website privacy statements
   d) type of website
   e) professional-looking website
   f) lock icon in the browser

## 2.2 App Installation

1) How frequently do you use an Android phone?
   a) Less than once a month
   b) Once a month
   c) Once a week
   d) Once a day
   e) Several times a day

2) How frequently do you install applications from the Android PlayStore? (Android PlayStore is where you search and install applications for your android based mobile phone)
   a) Never
   b) Every other month
   c) Once a month
   d) Once a week
   e) Every other day
   f) Every day
   g) Several times a day

3) How many applications have you installed from the Android PlayStore?
   a) 0
   b) 1-10
   c) 10-25
   d) 25+

4) Use the checkbox to select each of the options below in such a way that you select the most influential reason to install an application first and the least influential reason last (such that 1= Most influential reason for you to install an application and 8 = Least influential reason for you to install an application)
    a) Advertisement of the application
    b) Permissions requested is reasonable
    c) Rank of the application in the charts
    d) User reviews of the application
    e) Because my friends/family uses the application
    f) Popularity of the application
    g) Application features/benefits
    h) Design of the application

5) Do you review/read the permissions presented to you before you install an application from Android PlayStore?
    a) Yes
    b) No

6) How often do you review/read the permissions presented to you while you install an application from Android PlayStore?
    a) Never
    b) Sometimes
    c) Almost all the time
    d) Every time

7) How often have you stopped from proceeding to install an application because of the permissions requested by the application?
    a) Never
    b) Sometimes
    c) Almost all the time
    d) Every time

### 2.3 Demographics

1) What is the highest degree you have completed in school?
    a) Less than a high school
    b) High school
    c) Some college
    d) College Graduate
    e) Graduate School

2) What was your total household income from all sources before taxes last year?
    a) Less than $10,000
    b) $10,000 to $20,000
    c) $20,000 to $30,000
    d) $30,000 to $50,000
    e) $50,000 to $75,000

f) $75,000 to $100,000
g) $100,000 or more
h) Don’t know

3) What is your gender?
a) Female
b) Male
c) Other

4) What is your age?
a) Less than 18
b) 18-25
c) 25-35
d) 35-45
e) 45-55
f) 55-65
g) 65-75
h) 75+

## 3 Results

Here we report the results from our analysis on all participants that passed the attention check questions without filtering out participants for app installation time. The results were adjusted for multiple testing.

| | | p-values | Cohen’s d |
|---|---|---|---|
| Warning System Group | Dating Apps | 0.001 | 0.245 |
| | Puzzle Apps | P < 0.001 | 0.286 |
| Lock Group | Dating Apps | 0.010 | 0.148 |
| | Puzzle Apps | 0.009 | 0.156 |
| Sound Group | Dating Apps | 0.002 | 0.189 |
| | Puzzle Apps | 0.003 | 0.184 |

**Table S 1.** GEE results for Privacy Rating for data without the time filter with adjustments for multiple tests. These results show that participants in all experimental groups made app choices that are significantly different from that of the control group.

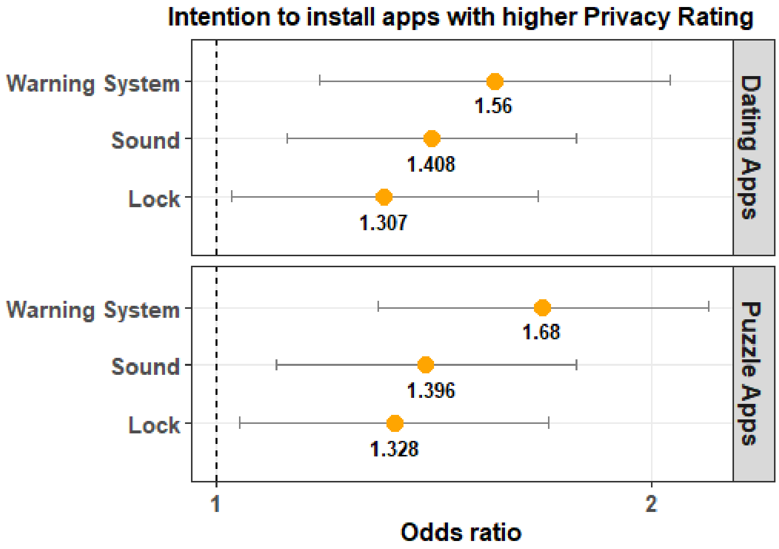


**Figure S 4.** The odds ratio (95% confidence interval) indicates that participants in all three experimental groups are more likely to select apps with a higher Privacy Rating. The effect size is larger for participants in the Warning System Group.

| | | p-values | Cohen's d |
|---|---|---|---|
| Warning System Group | Dating Apps | 0.002 | -0.252 |
| | Puzzle Apps | P < 0.001 | -0.232 |
| Lock Group | Dating Apps | 0.064 | -0.109 |
| | Puzzle Apps | 0.009 | -0.080 |
| Sound Group | Dating Apps | 0.183 | -0.034 |
| | Puzzle Apps | 0.003 | -0.010 |

**Table S 2.** GEE results for App Rating for data without the time filter. These results show that App Rating was statistically significant for the Warning System Group across both app categories. For the Lock and Sound Groups App Rating was only statistically significant for Puzzle Apps.

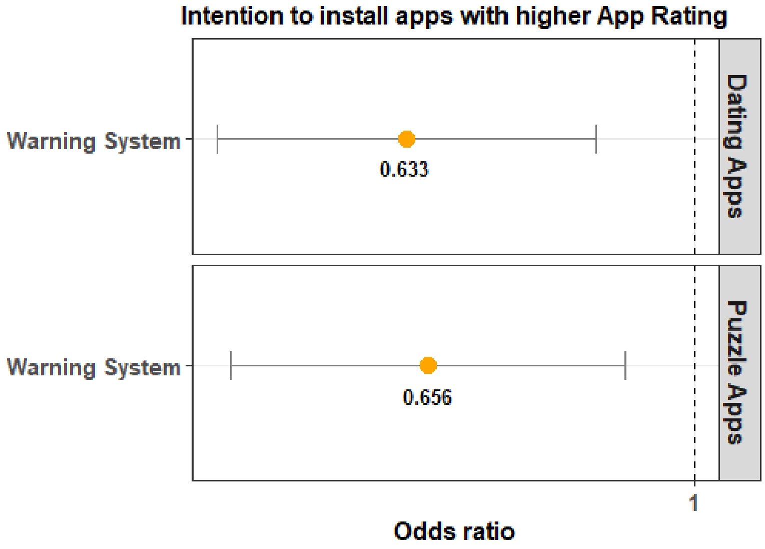


**Figure S 5.** The odds ratio (95% confidence interval) indicates that participants in the Control Group are more likely to select app with a higher App Rating when compared to the Warning System group for both app categories.

| | | p-values | Cohen's d |
|---|---|---|---|
| Warning System Group | Dating Apps | P < 0.001 | 0.308 |
| | Puzzle Apps | P < 0.001 | 0.334 |
| Lock Group | Dating Apps | 0.064 | 0.168 |
| | Puzzle Apps | 0.009 | 0.155 |
| Sound Group | Dating Apps | 0.183 | 0.125 |
| | Puzzle Apps | 0.003 | 0.124 |

**Table S 3.** GEE results for PrivacyOverAppRating for data without the time filter. These results show that PrivacyOverAppRating is statistically significant for the Warning System group and the Lock group. The results are not significant for the Sound group.

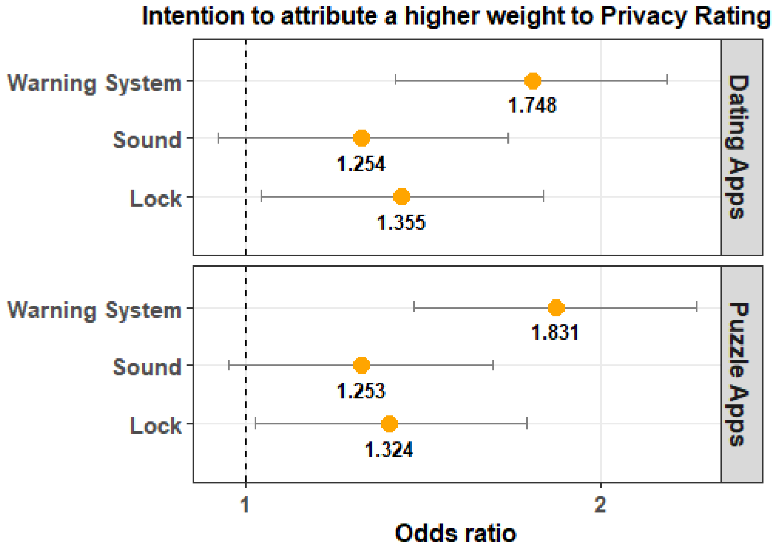


**Figure S 6.** The odds ratio (95% confidence interval) indicates that participants in the experimental groups are more likely to attribute a higher weight to Privacy Rating when compared to App Rating.

| | | p-values | Cohen's d |
|---|---|---|---|
| Warning System Group | Dating Apps | 0.002 | 0.184 |
| | Puzzle Apps | P < 0.001 | 0.228 |
| Lock Group | Dating Apps | 0.058 | 0.083 |
| | Puzzle Apps | 0.005 | 0.082 |
| Sound Group | Dating Apps | 0.002 | 0.194 |
| | Puzzle Apps | 0.010 | 0.151 |

**Table S 4.** GEE results for PrivacyOverDownloadCount for data without the time filter. These results show that PrivacyOverDownloadCount is statistically significant for Warning System Group across both app categories. For the Lock and Sound groups the results are only significant for puzzle apps.

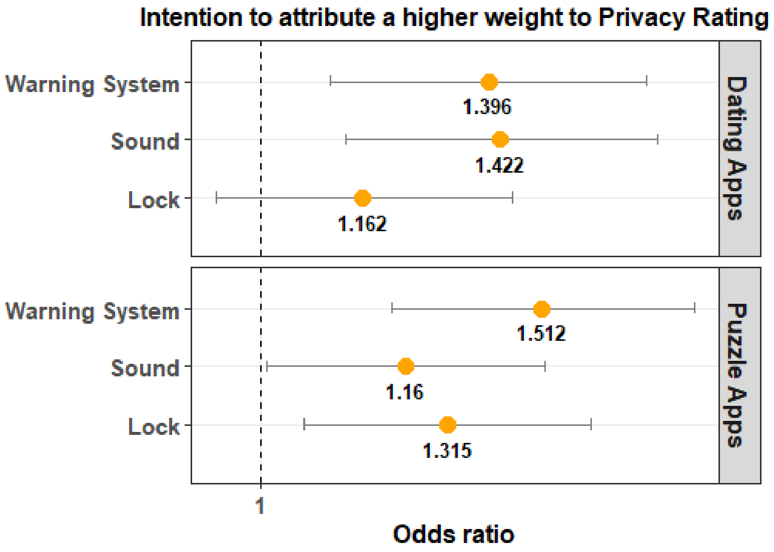


**Figure S 7.** The odds ratio (95% confidence interval) indicates that participants in the experimental groups are more likely to attribute a higher weight to Privacy Rating when compared to Download Count.
